# Nodakenin represses obesity and its complications via the inhibition of the VLDLR signalling pathway in vivo and in vitro

**DOI:** 10.1111/cpr.13083

**Published:** 2021-06-24

**Authors:** Bo‐Ram Jin, Minho Lee, Hyo‐Jin An

**Affiliations:** ^1^ Department of Pharmacology College of Korean Medicine Sangji University Wonju Korea; ^2^ Department of Life Science Dongguk University‐Seoul Goyang‐si Korea

**Keywords:** adipogenesis, Metainflammation, Nodakenin, obesity, oxidative stress, VLDLR

## Abstract

**Objectives:**

Nodakenin (NK) is a coumarin glucoside that is found in the roots of *Angelicae gigas*. A limited number of studies have been conducted on the pharmacological activities of NK. Although NK is an important natural resource having anti‐inflammatory and antioxidant effects, no investigation has been conducted to examine the effects of NK on obesity and obesity‐induced inflammation.

**Materials and Methods:**

The present study investigated the therapeutic effects of NK treatment on obesity and its complications, and its mechanism of action using differentiated 3T3‐L1 adipocytes and high‐fat diet (HFD)‐induced obese mice. Oil red O staining, western blot assay, qRT‐PCR assay, siRNA transfection, enzyme‐linked immunosorbent assay, H&E staining, immunohistochemistry, molecular docking and immunofluorescence staining were utilized.

**Results:**

Treatment with NK demonstrated anti‐adipogenesis effects via the regulation of adipogenic transcription factors and genes associated with triglyceride synthesis in differentiated 3T3‐L1 adipocytes. Compared with the control group, the group administered NK showed a suppression in weight gain, dyslipidaemia and the development of fatty liver in HFD‐induced obese mice. In addition, NK administration inhibited adipogenic differentiation and obesity‐induced inflammation and oxidative stress via the suppression of the VLDLR and MEK/ERK1/2 pathways. This is the first study that has documented the interaction between NK and VLDLR structure.

**Conclusion:**

These results demonstrate the potential of NK as a natural product‐based therapeutic candidate for the treatment of obesity and its complications by targeting adipogenesis and adipose tissue inflammation‐associated markers.

## Graphical abstract

Nodakenin (NK) regulated adipogenic differentiation and adipogenic transcription factors associated with triglyceride synthesis via inhibition of VLDLR in 3T3‐L1 adipocytes. Administration of NK ameliorated high fat‐diet (HFD)‐induced weight gain, dyslipidemia and the development of fatty liver in mice. NK administration suppressed adipogenic differentiation and obesity‐induced inflammation via the suppression of the VLDLR/VLDL and MEK/ERK1/2 pathways in HFD‐induced obese mice model. Inhibitory effect of NK is superior to that of Orlistat, highlighting the potential of NK as a therapeutic agent for treatment of obesity and its complications.

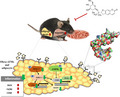

## 1 INTRODUCTION

Obesity is an important public health burden associated with an increased risk of metabolic disorders, including type 2 diabetes, non‐alcoholic fatty liver disease and cardiovascular diseases.^1^ Obesity is primarily characterized by adipocyte hypertrophy and hyperplasia and causes unusual metabolic inflammation. The energy surplus functions as a trigger of oxidative stress leading to chronic low‐grade inflammation or metaflammation, especially in metabolic tissues and organs such as white adipose tissue and liver, respectively.

In metabolic tissues, macrophages, directly or indirectly affected by oxidative stress, are major players in regulating metaflammation and obesity.^2^, ^3^, ^4^, ^5^ Several studies have highlighted the association between adipocytes and macrophages in obesity‐mediated complications.^6^ In an obese state, the expansion of the adipose tissue leads to the infiltration of adipose tissue macrophages (ATMs). Enhanced ATMs are the primary source of inflammatory responses. Stimulated ATMs induce cytokine secretion, which promotes the reciprocal release of free fatty acids from the adipocytes, forming a vicious loop.^7^

Adipocyte differentiation has been demonstrated in the 3T3‐L1 cell line, in which the cellular processes of adipogenesis were extensively characterized by three steps, namely, the arrest of the growth of confluent pre‐adipocytes, mitotic clonal expansion (MCE) and terminal differentiation. During MCE, cell proliferation and cell cycle progression occur in sequence. At the end of the cell cycle, the cells undergo terminal differentiation.^8^ The process of adipogenesis is regulated by the activation of the signalling pathways. During MCE, the activation of the signal transduction pathways, such as the PI3K/Akt pathways and mitogen‐activated protein kinase (MAPK) pathways including extracellular signal‐regulated kinase (ERK) and p38 MAPK, is involved. In addition, early adipogenic regulators, including CCAAT/enhancer‐binding protein (C/EBP) β and C/EBPδ, are transcriptionally activated during MCE. These transcription factors stimulate master adipogenic transcription factors fundamental to terminal differentiation, such as peroxisome proliferator‐activated receptors (PPAR) γ and C/EBPα.^9^ Meanwhile, it is also known that production of reactive oxidative stress (ROS) is markedly increased during adipocyte differentiation, indicative of the connection between ROS and adipogenesis.^10^

Recent evidence suggests that the expression of the very‐low‐density lipoprotein receptor (VLDLR) is regulated by PPARγ.^11^ VLDLR, a member of the low‐density lipoprotein receptor family, is highly expressed in the adipose tissue and plays a prominent role in lipid uptake and adipogenesis.^12^ There is convincing evidence that VLDLR is associated with obesity as demonstrated in humans and animal models.^13^, ^14^ In addition to adipocytes, VLDLR is also expressed in immune cells, including macrophages. Very low‐density lipoprotein (VLDL)‐VLDLR signalling in ATMs exacerbates adipose tissue inflammation in obesity, indicative of the role of VLDLR in adipose tissue inflammation and adipocyte‐macrophage interactions.^15^, ^16^

Although several medications are designed to treat obesity, most of them act as either appetite suppressants (such as sibutramine and amfepramone) or gastrointestinal lipase inhibitors (such as orlistat). Several agents have been subjected to clinical trials; however, only orlistat (Orli) and sibutramine were approved for long‐term drug therapy. However, the administration of these drugs results in certain adverse effects, including diarrhoea, tympanites, stomachache and severe liver injury.^17^ Hence, there is a need to develop substitutes based on a natural resource that could exert long‐term beneficial effects and reduce the side effects.

The natural product Nodakenin (NK), a furanocoumarin glycoside initially isolated from the roots of *Angelicae gigas* (Umbelliferae) has been reported to possess anti‐inflammatory, antioxidant and anti‐hyperglycaemic effects.^18^, ^19^, ^20^ There are a limited number of studies focusing on the biological effects of NK, except those demonstrating its anti‐inflammatory effects.^21^, ^22^ Studies using macrophage cells and animal models have reported that NK exhibits anti‐inflammatory effects.^23^, ^24^ Our previous study also demonstrated the mechanisms responsible for the anti‐allergic inflammatory activity of NK.^25^ However, the effect of NK on obesity and obesity‐induced inflammation has not yet been documented. Therefore, to evaluate the therapeutic effects of the NK on obesity and its complications, we investigated the molecular mechanism(s) underlying the anti‐adipogenic, anti‐inflammatory and antioxidant effects of NK in 3T3‐L1 cells and a high‐fat diet (HFD)‐induced obesity mouse model.

## 2 MATERIAL AND METHODS

### 2.1 Chemicals and reagents

3‐(4,5‐dimethylthiazol‐2‐yl)‐2,5‐diphenyltetrazolium bromide (MTT), 3‐Isobutyl‐1‐methylxanthine (IBMX), dexamethasone (DEX), insulin, Oil Red O powder and all other chemicals were purchased from Sigma‐Aldrich Co. LLC. Dulbecco's modified Eagle's medium (DMEM), bovine serum, foetal bovine serum and antibiotic‐antimycotic were obtained from Life Technologies, Inc. NK was purchased from ChemFaces (Hubei). Orlistat was acquired from TCI. The primary antibodies against sterol regulatory element‐binding transcription factor (SREBP)‐1 (Cat.no. sc‐13551), PPARγ (Cat.no. sc‐7273), C/EBPα (Cat.no. sc‐9314), VLDLR (Cat.no. sc‐18824), CD68 (Cat.no. sc‐20060), mitogen‐activated protein kinase kinase (MEK)1/2 (Cat.no. sc‐17820), p‐ ERK1/2 (Cat.no. sc‐7383), F4/80 (Cat.no. sc‐377009) and β‐actin (Cat.no. sc‐47778) were purchased from Santa Cruz Biotechnology, Inc. Antibodies against 5′ AMP‐activated protein kinase (AMPK) (Cat.no. #2532), p‐AMPK (Cat.no. #2535), P‐MEK1/2 (Cat.no. #2535) and ERK (Cat.no. #9101), c‐Fos (Cat.no. #2250), were acquired from Cell Signaling Technology (MA, USA). Peroxidase‐conjugated secondary antibodies were purchased from Jackson ImmunoResearch, Inc Primers corresponding to SREBP1, SREBP2, PPARγ, C/EBPα, C/EBPβ, fatty acid synthase (FAS), HMG‐CoA, adipocyte fatty acid‐binding protein 2 (aP2), liver X receptor (LXR), low‐density lipoprotein receptor (LDLR), VLDLR and Glyceraldehyde 3‐phosphate dehydrogenase (GAPDH) oligonucleotide were purchased from Bioneer Corporation, and SYBR Premix Ex Taq was purchased from Takara Bio Inc. Mounting medium with 4,6‐diamidino‐2‐phenylindole (DAPI) was got from Vector Laboratories, Inc. Fluorescein isothiocyanate (FITC) conjugated anti‐mouse and tetramethylrhodamine (TRITC) conjugated anti‐rabbit secondary antibodies were obtained from Invitrogen Corp.

### 2.2 Cell culture and differentiation

3T3‐L1 pre‐adipocytes were obtained from the Korean Cell Line Bank and cultured in DMEM with 10% bovine serum, sodium carbonate, HEPES and 1% antibiotic/antimycotic. For the induction of adipocyte differentiation, 3T3‐L1 pre‐adipocytes (lines <8) were cultured until confluence (0 days) and the culture media was replaced with differentiation‐inducing media containing 10% foetal bovine serum and differentiation medium cocktail (0.5 mmol/L IBMX, 1 μmol/L DEX and 5 μg/mL insulin) for 2 days. The media was then replaced with DMEM containing 5 μg/mL insulin and 10% FBS. After 2 days, the media was changed with a fresh DMEM with 10% FBS. This step was repeated every 2 days until the cells were harvested. To evaluate the inhibitory effect of NK on adipogenic differentiation, seeded cells were treated with NK on days 0, 2, 4 and 6. Cells were cultured in a humidified environment with 5% CO_2_ at 37℃.

### 2.3 Cell viability

Cells were treated with NK (0‐500 μmol/L) and incubated overnight. Next, the MTT solution (5 mg/mL) was added for 2 hours. After soaking off the supernatant, the formazan product was dissolved in DMSO, and the extent of cytotoxicity was measured at 570 nm using a BioTek™ Epoch microplate spectrophotometer (Biotek). Experiments were performed in triplicate in a parallel manner and the values were represented as mean ± standard deviation (SD).

### 2.4 Oil red O staining

Fully differentiated 3T3‐L1 cells were washed twice with phosphate‐buffered saline and fixed with 10% formalin for 1 hour. After fixation, the cells were washed thrice with distilled water and then stained with 0.4% Oil Red O solution in 60:40 (v/v) isopropanol/ H_2_O for 2 hour at room temperature. The cells were washed with distilled water and observed under a Leica microscope (Leica DFC295). The Oil Red O dye was eluted using 100% isopropanol and the absorbance was measured at 490 nm using a microplate reader (Biotek).

### 2.5 Real‐time quantitative reverse transcription PCR (qRT‐PCR) assay

After homogenization of the cells and tissues, total RNA was isolated with Easy‐Blue Reagent (Intron Biotechnology Inc.) according to the manufacturer's instructions. Quantification of total RNA was performed with an Epoch microvolume spectrophotometer system (BioTek Instruments Inc.). cDNA was obtained using isolated total RNA (1 µg), d(T)16 primers and AMV reverse transcriptase. Relative gene expression was quantified using real‐time PCR technique (Real‐Time PCR System 7500, Applied Biosystems) with SYBR Green PCR Master Mix (Applied Biosystems) and oligonucleotide primers (Table 1) purchased from Bioneer. The threshold cycle (Ct) values of genes were normalized to the Ct values of GAPDH using the gene express 2.0 program (Applied Biosystems). We used the comparative Ct method to calculate fold changes in gene expression.

**TABLE 1:** Primer sequences

Gene name	Forward primers (5′‐3′)	Reverse primers (5′‐3′2)
SREBP‐1	TCAAGGCACATTTTTGCTCC	ATCGCAAACAAGCTGACCTG
SREBP‐2	TGCTGGATGACGCAAAGGTC	AAAGGAGAGGCCCAGGAAGG
PPAR*γ*	TTCGGAATCAGCTCTGTGGA	CCATTGGGTCAGCTCTTGTG
C/EBP‐α	TCGGTGCGTCTAAGATGAGG	TCAAGGCACATTTTTGCTCC
C/EBP‐β	GGGGTTGTTGATGTTTTTGG	CGAAACGGA AAAGGTTCTCA
FAS	GCTGCAAGCACAGCCTCTCT	GGCATCATTGGGCACTCCTT
HMG‐CoA	TGGCAGAAAGAGGGAAAGG	CGCCTTTGTTTTCTGGTTGA
Ap2	AGCATCATAACCCTAGATGG	GAAGTCACGCCTTTCATAAC
LXR	AGATCCAGGTTTGAGGTGGG	TCCTACACGAGGATCAAGCG
LDLR	CTCACTTCCGCTGCAACTCC	CCACAGTGGAACTCGAGGGA
VLDLR	TCCAATGGCCTAATGGAATTACA	AGCATGTGCAACTTGGAATCC
GAPDH	TGATTCTACCCACGGCAAGT	AGCATCACCCCATTTGATGT

### 2.6 Western blot analysis

The mice liver and adipose tissues as well as 3T3L‐1 cells were homogenized in a commercial lysis buffer PRO‐PREP (Intron) and centrifuged at 15928*g* (4℃) for 5 minutes to extract protein. Subsequently, the supernatant was transferred to a fresh 1.5 mL tube. The quantification of the protein was carried out using Bio‐rad protein assay reagent (Bio‐Rad Laboratories, Inc.). Quantified samples (30 μg) in each lane were fractionated on an 8%‐12% gradient SDS gel, transferred to Polyvinylidene fluoride (PVDF) membranes, and probed with specific primary antibodies in T‐TBS (2.5% skim milk) solution at 4°C, followed by incubation with peroxidase‐conjugated secondary antibodies (Jackson ImmunoResearch) at 25°C. Bands were visualized using the ECL solution (Ab signal) on an X‐ray film (Agfa, Belgium). Western blotting experiments were performed in triplicate, and data were quantified and expressed as means ± SDs in relative protein level graph.

### 2.7 Molecular docking

To predict the binding of NK on VLDLR, molecular docking was carried out using Autodock Vina.^26^ The structure of NK was downloaded from PubChem (CID: 73191). The value of exhaustiveness in AutoDock Vina was set to 40. NK‐bound structure of VLDLR was visualized and analysed by PyMol^27^ and two‐dimensional interactions between NK and VLDLR were estimated by LigPlot+.^28^

### 2.8 VLDLR transfection

The fully differentiated 3T3‐L1 cells received VLDLR siRNA (Bioneer Corporation, Table 2) or pmaxGF0050™ vector using 4D‐Nucleofector™ system (Lonza). After 4 hours incubation, the cells were treated with the presence and absence of NK100. The impact of VLDLR knockdown transfection was detected via qRT‐PCR assay.

**TABLE 2:** Nucleotide sequence of VLDLR siRNA

SiRNA	Forward sequence	Reverse sequence
VLDLR siRNA 1	CUGUUGAUUGGGUGUACAA = tt(1‐AS)	UUGUACACCCAAUCAACAG= tt(1‐AA)
VLDLR siRNA 2	CUCUCUUGCUCUUAGUGAU = tt(2‐AS)	AUCACUAAGAGCAAGAGAG = tt(2‐AA)
VLDLR siRNA 3	CGAGGUUUGCAAAGACUGA = tt(3‐AS)	UCAGUCUUUGCAAACCUCG = tt(3‐AA)

### 2.9 Animal

Male C57BL/6N mice (n = 48; 8 weeks old; 18‐22 g; Daehan Biolink) were housed 10 per cage in the animal room with 12 hours dark/light cycles and constant temperature (temperature, 20 ± 5℃; humidity, 40%‐60%) with free access to food and tap water. All animal studies were performed in accordance with the guidelines for the care and use of laboratory animals established by the National Institutes of Health. The Institutional Animal Care and Use Committee (IACUC) of the Sangji University certified and approved all animal experimental protocols (no. 2017‐23).

### 2.10 Induction of obesity and drug administration

Using a blinded method, mice were randomly distributed into six groups (n = 8 per cage): the normal diet group (CON), 45% HFD group, orlistat‐administered groups (Orli 10 and 20 mg/kg orally^21^) and NK‐administered groups (NK 10 and 20 mg/kg p.o.). Except for animals belonging to the normal group, all animals were fed with HFD. The normal diet consisted of a standard laboratory chow (NIH‐41 open formula diet; Zeigler Bros., Inc.) with 5% fat, whereas the HFD contained 45% fat (D12451 open formula diet; Research Diets, Inc.). After 8 weeks of high‐fat diet feeding, orlistat or NK treatment groups were administered the respective treatments orally, whereas the other groups were treated with physiological saline. Bodyweight and food intake were recorded every week. At the end of 13 weeks, all animals were fasted for 12 hours and anaesthetized with Zoletil 50 (20 mg/kg; Virbac, Carros Cedex) administered intraperitoneally according to the manufacturer's instruction. The liver and adipose tissues were harvested, rinsed, weighed and directly stored at −80℃ until further analysis.

### 2.11 Serum analysis

During blood sample collection, animals were already under the influence of terminal anaesthesia. Blood samples were collected by cardiac puncture. The samples were centrifuged at 1005 *g* for 10 minutes to obtain serum samples. The concentrations of aspartate aminotransferase (AST), alanine aminotransferase (ALT), blood urea nitrogen (BUN), triglyceride and total cholesterol were measured by enzymatic methods with a mercantile available assay kit (BioVision Research Products, Inc.).

### 2.12 Histological analysis

The liver and adipose tissues collected from each group were fixed with 4% formalin and embedded in paraffin. The tissues were cut into 4‐mm sections. The sections were stained with haematoxylin and eosin (H&E) for histological examination. Images were acquired with a Leica microscope (Leica DFC295).

### 2.13 Immunohistochemistry (IHC)

All IHC experiments were performed with the formalin‐fixed, paraffin‐embedded sample. Paraffin blocks were cut into 5 μm thick sections, mounted onto poly‐L‐lysine‐coated slides, and dried. After the dried slides were de‐paraffinized, antigen retrieval was performed for 20 minutes by using an automated antigen retrieval machine in the presence of ethylenediaminetetraacetic acid (pH 9.0). Non‐specific binding to the sections was blocked by incubation in 10% normal goat serum (Gibco Life Technologies) for 1 hour prior to incubation with the VLDLR (Cat.no. sc‐18824) and iNOS (Cat.no. sc‐650) primary antibodies overnight at 4℃. Secondary mouse antibodies were used to detect primary antibodies, followed by incubation with streptavidin‐tagged horseradish peroxidase (Ventana Medical Systems). Diaminobenzidine (Sigma‐Aldrich) was used to induce signalling. The IHC slides were visualized with an optical microscope (Leica) and analysed with Leica software.

### 2.14 VLDL assay

The levels of VLDL in adipose tissue were quantified with mouse VLDL assay Kit (DoGenBio) according to the manufacturer's handbook.

### 2.15 Immunofluorescence (IF) staining

After deparaffinization and rehydration, slides were incubated with anti‐mouse VLDLR antibodies and anti‐rabbit iNOS antibodies and visualized with FITC conjugated anti‐mouse and TRITC conjugated anti‐rabbit secondary antibodies, respectively. Slides were mounted and detected with a Nikon X‐cite series 120 Q microscope (Nikon). The exposure parameters were kept the same for each sample.

### 2.16 Statistical analysis

Experiments were performed in triplicate, and data are expressed as mean ± SD. Statistically significant values were determined using analysis of variance (ANOVA) and Dunnett's post hoc test. *P*‐values ˂ .05 were considered statistically significant. Statistical analysis was performed using GraphPad Prism 5.

## 3 RESULTS

### 3.1 NK inhibits droplet accumulation and adipogenic differentiation in 3T3‐L1 cells

**FIGURE 1:**
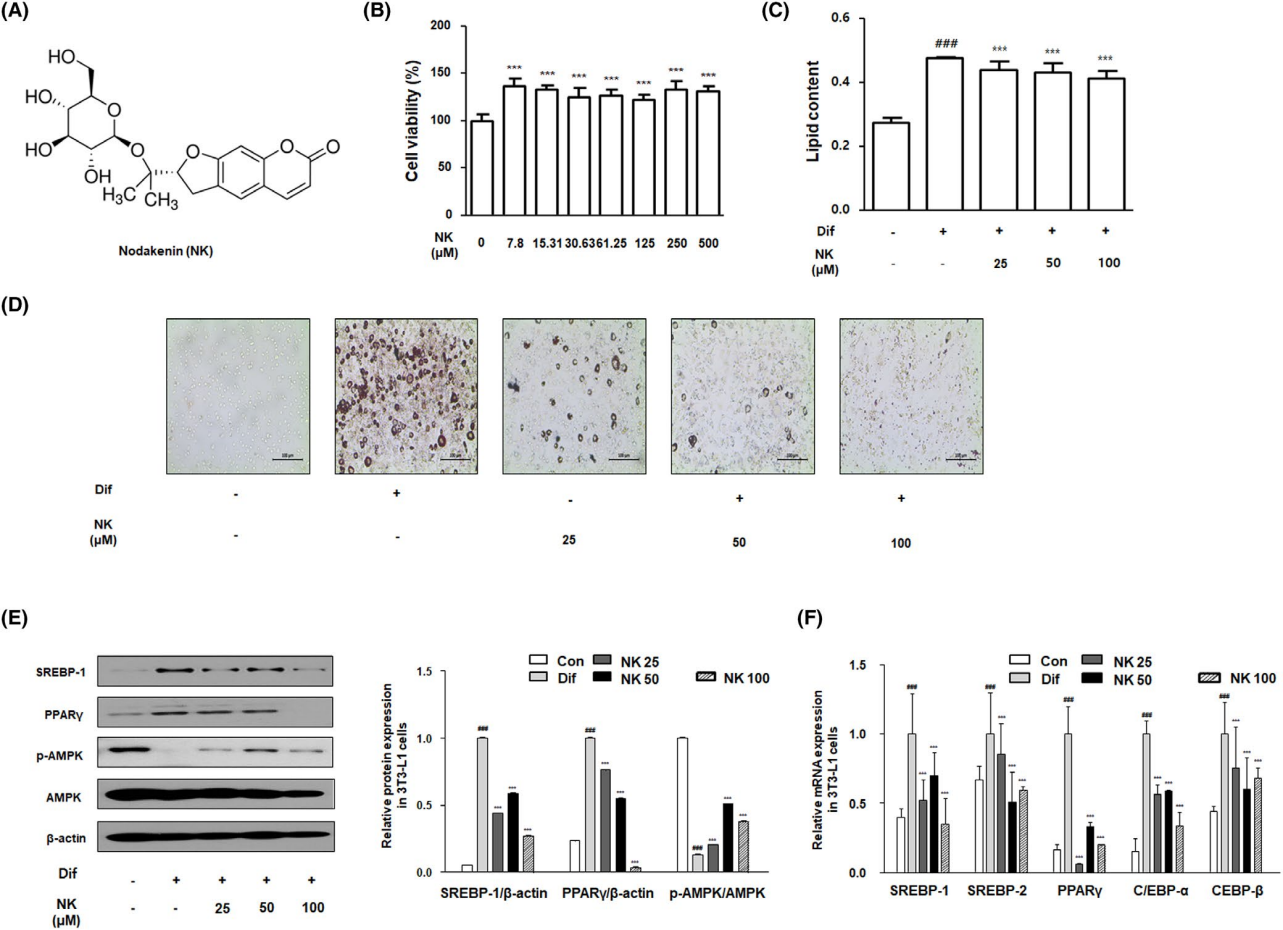
Effect of NK on lipid accumulation mediated via the regulation of adipogenic factors in 3T3‐L1 cells. A, The chemical structure of NK (B) Results of the MTT assay for the detection of the cytotoxicity of NK on 3T3‐L1 cells. Experiments were performed in triplicate parallelly and the values were represented as mean ± SD. C and D, Results of Oil red O staining to determine lipid accumulation with the presence and absence of NK in 3T3‐L1 cells. The stained cells were visualized using a microscope at 100× magnification. E, The protein levels of SREBP‐1, PPARγ, p‐AMPK, AMPK in 3T3‐L1 cells. F, The mRNA level of adipogenic factors in 3T3‐L1 cells. Values are the mean ± SD; ^###^ *P* < .001 vs the control group; ***P < .001 vs groups with differentiation‐inducing conditions; Significant differences between NK‐treated groups were determined using ANOVA and Dunnett's post hoc test

**FIGURE 2:**
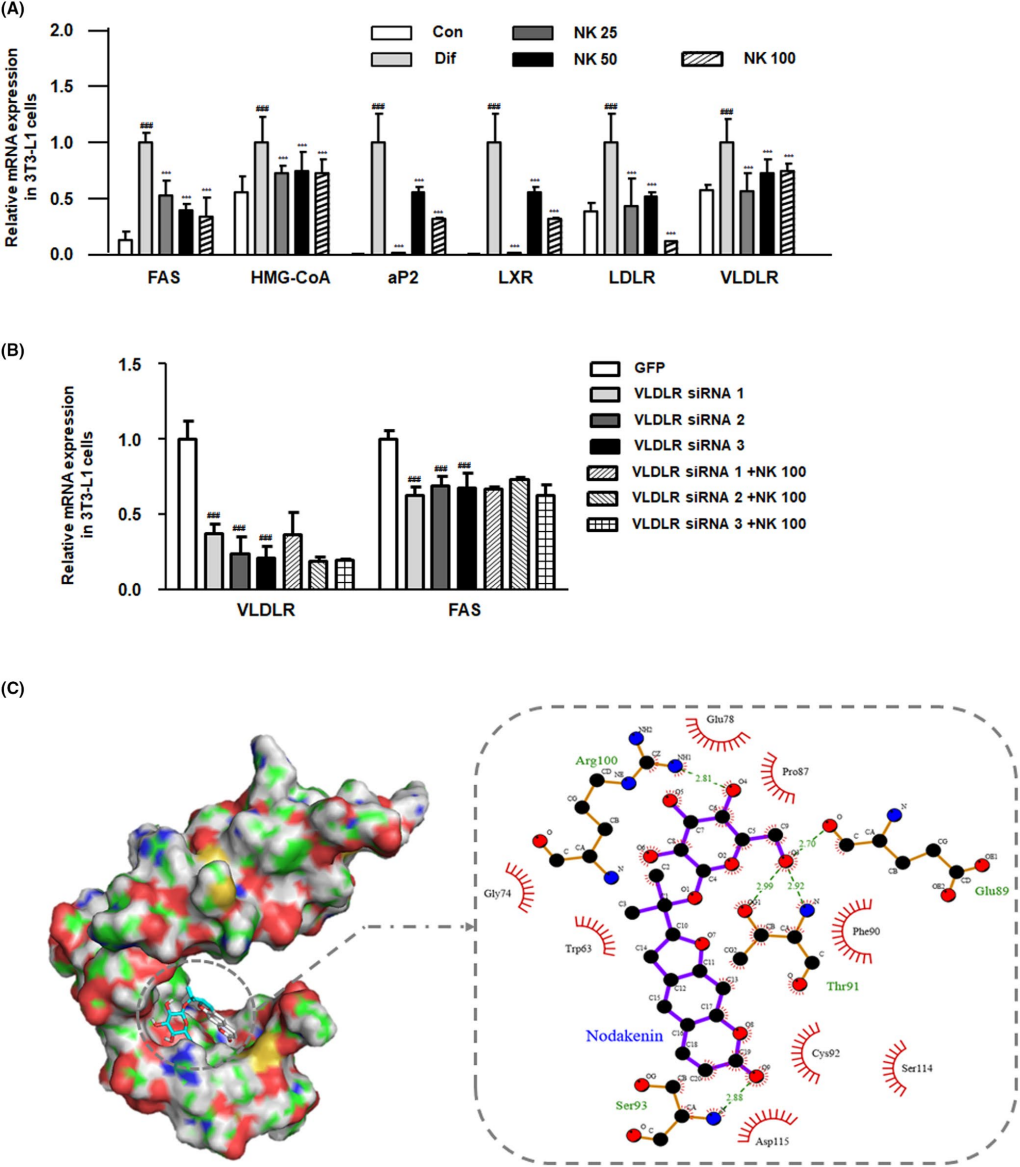
Effect of NK on VLDLR signalling in 3T3‐L1 cells. A, The mRNA level of FAS, HMG‐CoA, aP2, LXR, LDLR and VLDLR in 3T3‐L1 cells. Values are the mean ± SD; ^###^ *P* < .001 vs the control group; ***P < .001 vs groups with differentiation‐inducing conditions; Significant differences between NK‐treated groups were determined using ANOVA and Dunnett's post hoc test. B, The mRNA level of VLDLR and FAS in 3T3L1 cells transfected with VLDLR siRNA. Values are the mean ± SD; ^###^ *P* < .001 vs the GFP group. C, Molecular docking simulation to analyse the interaction between NK and VLDLR structure using the Autodock Vina program

To investigate the effect of NK (Figure 1A) on cell viability in 3T3‐L1 cells, 3T3‐L1 preadipocytes were incubated with various concentrations of NK for 24 hours. The results showed that NK did not lead to toxicity in 3T3‐L1 preadipocytes (Figure 1B). To evaluate the inhibitory effect of NK on adipocyte differentiation, 3T3‐L1 cells were cultured with differentiation media. 8 days after treatment with the differentiation media along with the indicated concentrations of NK, Oil red O staining used to assess the effect of NK on the accumulation of intracellular lipid droplets in 3T3‐L1 cells. As shown in Figure 1C and 1D, the Oil red O staining demonstrated that the accumulation of lipid droplet significantly increased under differentiation‐inducing conditions than that under control conditions, whereas NK suppressed the lipid droplet accumulation in a concentration‐dependent manner. Subsequently, we studied relative gene expression profiles to evaluate the effect of NK on lipid metabolism. As shown in Figure 1E, the differentiation‐inducing conditions increased the protein expression of SREBP‐1 and PPARγ and decreased the phosphorylation of AMPK. NK significantly regulated the change in the differentiation‐induced protein expression. Consistent with the protein expression results, mRNA expression data demonstrated that NK significantly suppressed SREBP‐1 and PPARγ mRNA compared to that in the differentiation condition group. Next, we determined the mRNA expression of other genes associated with adipocyte differentiation and lipid biosynthesis. Similar to the results of SREBP‐1 and PPARγ mRNA expression, NK treatment suppressed the mRNA expression of SREBP‐2, CEBP‐α, CEBP‐β, FAS, HMG‐CoA, Ap2, LXR, LDLR and VLDLR in contrast to gene expression under differentiation‐inducing conditions (Figures 1F and 2A).

### 3.2 VLDLR inhibition contributes to the impact of NK on adipogenesis in 3T3‐L1 cells

Very low‐density lipoprotein receptor is absent in preadipocytes but is remarkably manifested during adipogenesis and actively participates in adipocyte hypertrophy.^16^ Figure 2A demonstrates that NK treatment suppressed the VLDLR mRNA expression in the 3T3‐L1 adipocytes. To demonstrate the role of VLDLR pathway in the effect of NK, differentiated 3T3‐L1 cells were transfected with VLDLR siRNA and treated with NK100 for 12h. As shown in Figure 2B,A successful transfection was carried out as the transfected 3T3L1 cells show the lower expression of VLDLR mRNA as compared to the GFP group. We next examined if VLDLR is responsible for the effects of NK on 3T3‐L1 cells and realized that the knockdown of VLDLR diminished the inhibitory effects of NK on VLDLR and FAS mRNA expression compared to the GFP group. Next, a structure‐based molecular docking analysis was performed to identify the interactions between VLDLR and NK. The results indicated that NK interacts with VLDLR forming hydrogen bonds in Glu89, Thr91 and Arg100, concurrently (Figure 2C).

### 3.3 NK suppresses weight gain in the HFD‐induced obesity mouse model

**FIGURE 3:**
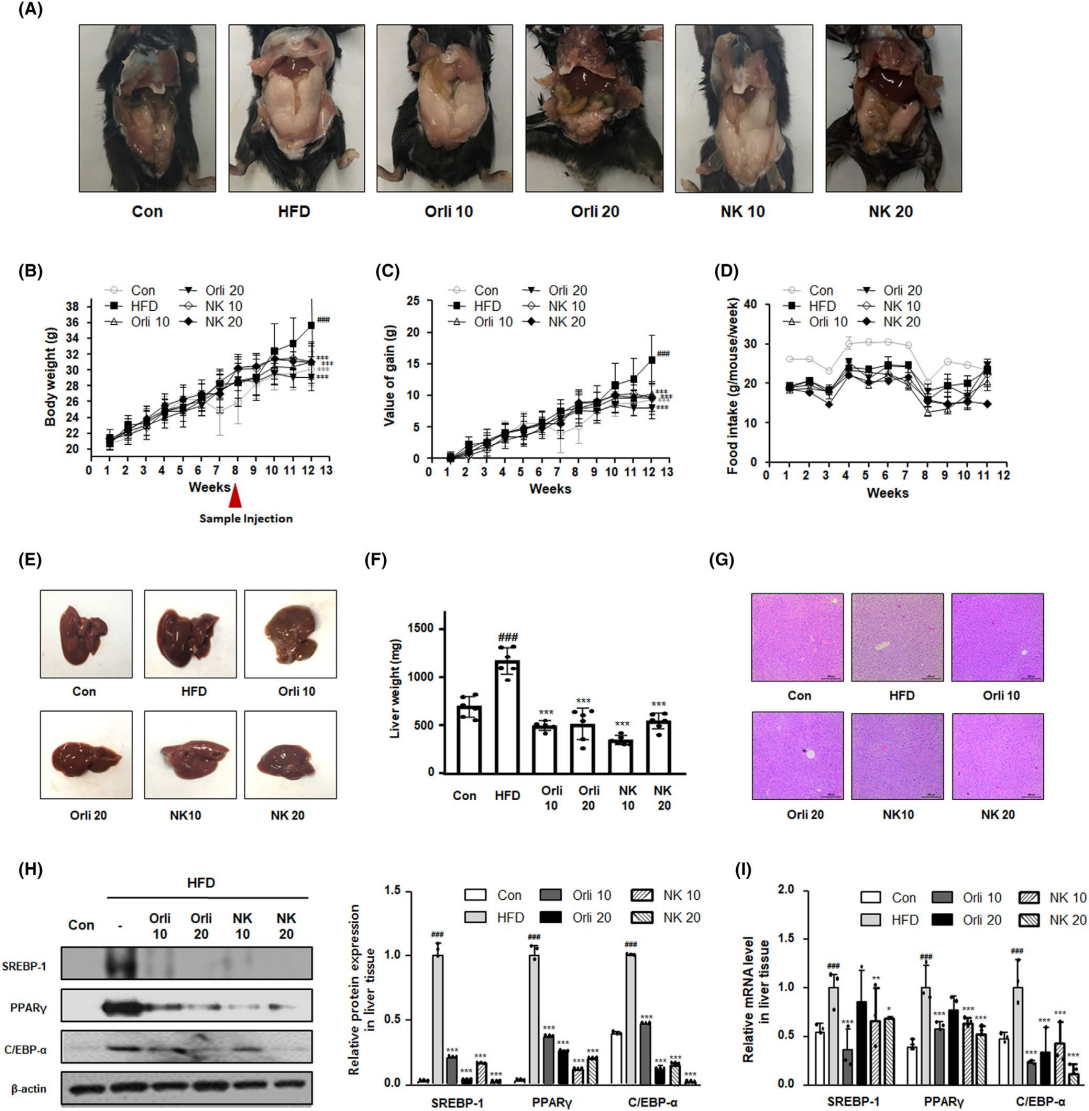
Effect of NK on weight gain and hepatic fat accumulation in mice with HFD‐induced obesity. A, Photograph of the mouse body and epidydimal adipose tissues in the HFD‐induced obesity model. B, Body weight and (C) weight gain values were assessed every week. D, Food intake was recorded two or three times per week. E, A representative photograph of liver tissues in each experimental group (F) Liver weight was measured in each experimental group. G, Representative image of H&E staining of liver tissues from each group. Original magnification 100×. H, The protein levels of adipogenic factors in liver tissues. The total protein lysates were prepared and evaluated for determining the levels of SREBP‐1, PPARγ and C/EBP‐α by western blot analysis using specific antibodies. Relative ratio level normalized to β‐actin was determined by densitometric analysis (Bio‐rad Quantity One^®^ Software) (I) mRNA expression of adipogenesis‐related genes (SREBP‐1, PPARγ and C/EBP‐α) in liver tissues were analysed by the qRT‐PCR assay. The values are expressed as mean ± SD of 8 mice per group. ^###^ *P* < .001 vs the Con group; *P < .05, **P < .01, ***P < .001 vs HFD group

To investigate the therapeutic effects of NK on obesity, we established the HFD‐induced obesity mouse model as described in the methods section. After 13 weeks, the HFD mice exhibited an increase in fat pad compared with the mice fed with a normal diet, whereas Orli20 and NK20 administration in mice alleviated the overproduction of fat pad induced by HFD (Figure 3A). As shown in Figure 3B,C, mice in the HFD group demonstrated a significantly increased body weight and weight gain values compared to mice in the control group. In contrast with the HFD group, treatment with Orli10, Orli20, NK10 and NK20 significantly attenuated the weight gain by 34.53%, 48.80%, 37.45% and 38.72%, respectively. No significant difference was observed in food intake among all experimental groups for 13 weeks (Figure 3D).

### 3.4 NK suppresses HFD‐induced dyslipidaemia in the HFD‐induced obesity mouse model

Animals fed a HFD demonstrated a significant dyslipidaemic plasma profile, with high levels of AST, ALT, BUN, triglyceride and total cholesterol. As shown in Table 3, after 5 weeks of treatment with Orli20 and NK20, the AST levels decreased significantly compared to that of the HFD group. In addition, NK20 treatment notably suppressed the upregulation of ALT, indicating the hepatoprotective role of NK. Following the induction of obesity, BUN levels significantly increased up to 1.52 times as compared to that in the control group, whereas the administration of Orli and NK significantly reduced the levels of BUN. The HFD group demonstrated abnormally high levels of triglyceride compared to mice in the control group. In contrast, the administration of NK alone significantly suppressed the upregulation of triglyceride. Meanwhile, treatment with Orli and NK did not demonstrate a significant change in total cholesterol levels. These results showed that the administration of NK suppresses HFD‐induced hyperlipidaemia in mice.

**TABLE 3:** Effect of NK on plasma biochemistry in mice with HFD‐induced obesity

Parameters Groups	ALT (IU/L)	AST (IU/L)	BUN (mg/dL)	Triglyceride (mg/dL)	Total cholesterol (mg/dL)
Con	7.66 ± 4.06	23.92 ± 2.13	24.50 ± 1.87	87.83 ± 8.33	102.33 ± 3.20
HFD	18.39 ± 4.68^###^	46.47 ± 5.37^###^	39.83 ± 6.15^###^	113.17 ± 3.30^##^	151.17 ± 11.70^###^
Orli10	18.50 ± 2.36	35.21 ± 6.71	24.17 ± 3.13***	108.83 ± 7.47	152.50 ± 6.92
Orli20	15.68 ± 4.19	24.52 ± 4.85	28.33 ± 2.80***	113.00 ± 2.43	156.50 ± 8.50
NK10	17.09 ± 2.52	34.24 ± 3.60	21.50 ± 1.38***	78.17 ± 7.28***	148.50 ± 7.50
NK20	3.97 ± 1.81**	26.37 ± 3.57***	24.50 ± 3.39***	72.33 ± 7.50***	146.67 ± 6.15

Note

ALT, AST, BUN, triglyceride and total cholesterol levels were estimated in mice with HFD‐induced obesity. The values are expressed as mean ± SD of 8 mice per group.

^##^ *P* < .01.

^###^ *P* < .001 vs the Con group.

** *P* < .01

*** *P* < .001 vs HFD group; significant differences between the treatment groups were determined using ANOVA and Dunnett's post hoc test.

### 3.5 NK represses the development of fatty liver in the HFD‐induced obesity mouse model

Long‐term consumption of HFD not only leads to weight gain but also exacerbates the development of fatty liver, leading to several metabolic complications, which may be linked to oxidative stress.^29^ Consistent with serum profiles, the administration of NK also demonstrated beneficial effects on lipid metabolism in liver tissues. As seen from Figure 3E,F, mice with obesity had a notable colour change that may represent the accumulation of a high amount of fat and increased liver weight compared to that in the control group. In contrast with mice in the HFD group, Orli and NK treatments resulted in a significantly reduced liver weight. Histological examination of the liver demonstrated that mice in the HFD group exhibited lipid droplet accumulation compared to those in the control group. However, the treatment with Orli and NK reduced HFD‐induced lipid production in liver tissues (Figure 3G). In addition, Orli and NK treatments significantly suppressed the relative protein and mRNA levels of SREBP‐1, PPARγ and CEBP‐α in the liver tissues compared to that in the HFD group (Figure 3H,I). These results indicate that the administration of NK exhibits therapeutic effects alleviating HFD‐induced hepatic steatosis.

### 3.6 NK inhibits enlargement of adipocytes in the HFD‐induced obesity mouse model

**FIGURE 4:**
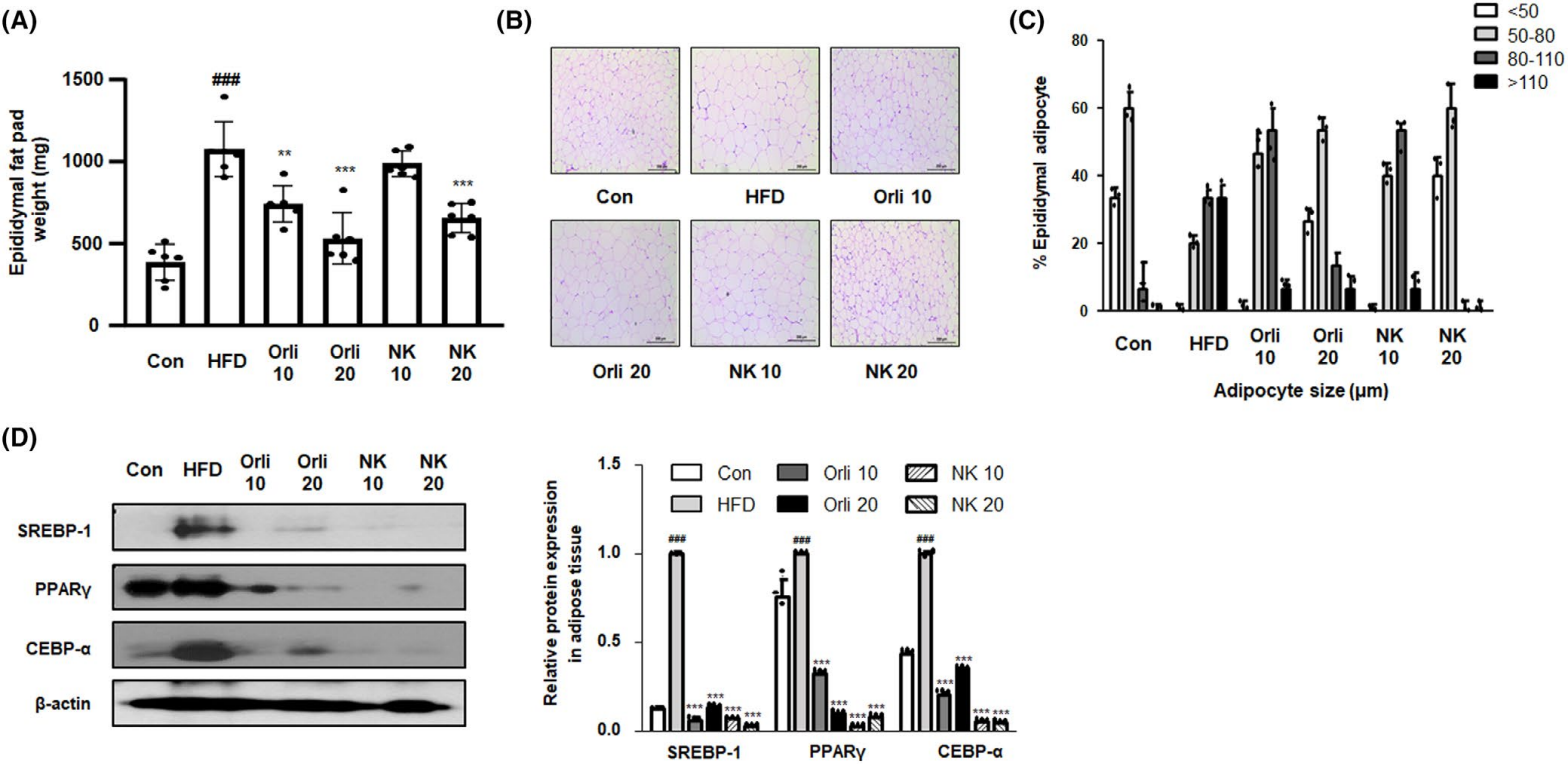
Effect of NK on adipogenesis and fat accumulation in mice with HFD‐induced obesity. A, The weight of the epididymal fat pad was measured in each experimental group. B, H&E staining analysis was performed using epididymal adipose tissue sections. Original magnification 100×. C, Based on H&E staining, adipocyte size was measured. D, The protein level of SREBP‐1, PPARγ and C/EBP‐α in adipose tissue was estimated by western blot analysis. Relative ratio level normalized to β ‐actin was determined by densitometric analysis (Bio‐rad Quantity One^®^ Software). All data are shown as the average value of each experimental group and are represented as mean ± SD (n = 8). ^###^ *P* < .001 vs the Con group; **P < .01, ***P < .001 vs HFD group

As shown in Figure 4A, mice in the HFD group demonstrated a significantly increased epididymal fat weight compared to mice in the control group. In contrast, the treatment with Orli and NK significantly alleviated the overproduction of the epididymal fat pad. Previous studies have demonstrated that a HFD induces either hypertrophy or hyperplasia in adipocytes.^30^ H&E staining demonstrated that the HFD diet increased the adipose size and number in epididymal fat tissues, whereas the administration of Orli and NK suppressed HFD‐induced adipocyte hypertrophy or hyperplasia (Figure 4B,C). Furthermore, the protein expression of major adipogenic trans‐factors, including SREBP‐1, PPARγ and CEBP‐α in the epididymal adipose tissues increased in the HFD group compared to the control group, whereas the administration of Orli and NK significantly inhibited the overexpression of SREBP‐1, PPARγ and CEBP‐α (Figure 4D).

### 3.7 NK inhibits adipogenesis via suppression of VLDLR in macrophages in the HFD‐induced obesity mouse model

**FIGURE 5:**
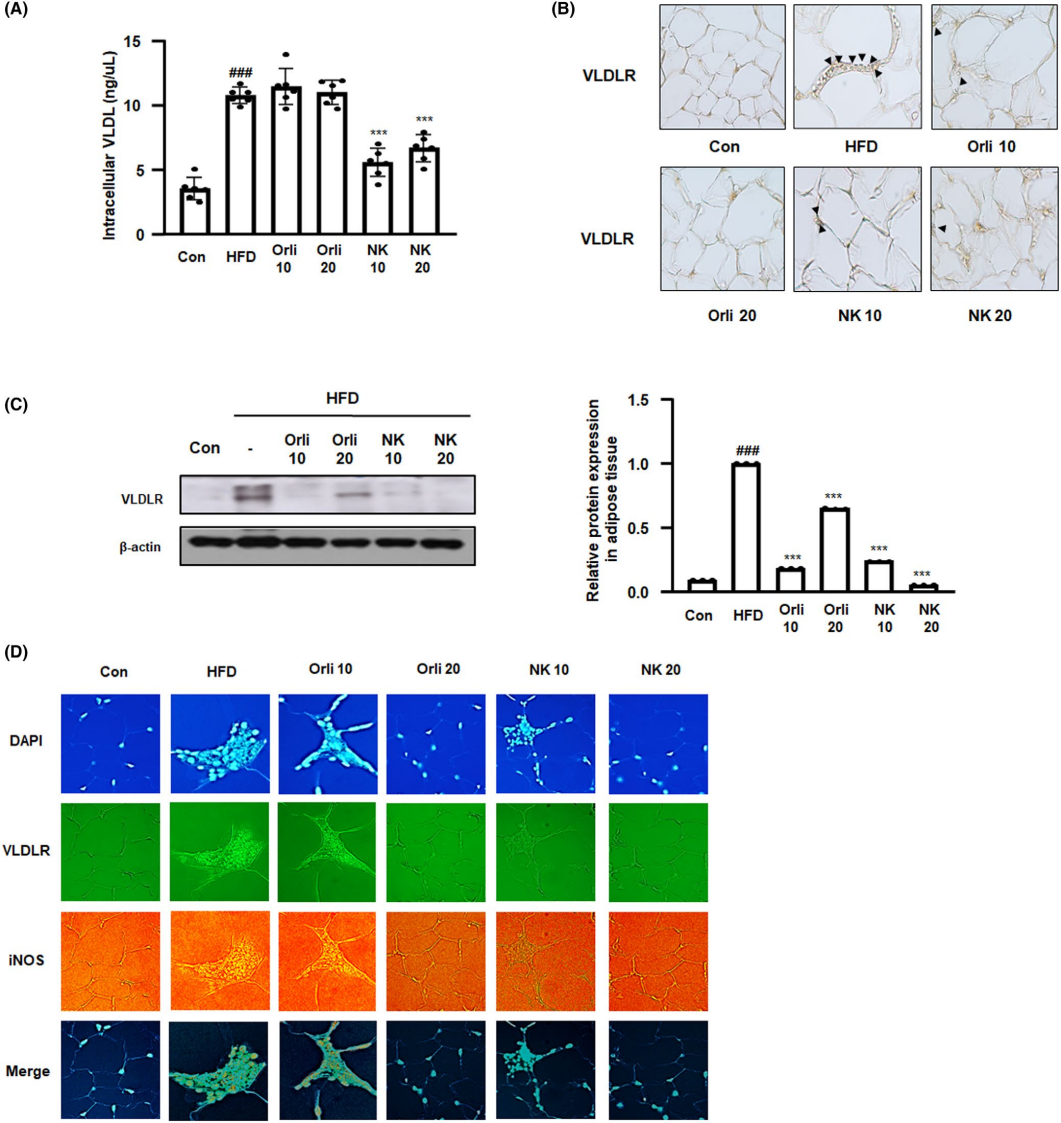
Effect of NK on VLDL/VLDLR signalling in mice with HFD‐induced obesity. A, The concentration of VLDL in adipose tissues was determined by enzyme immunoassay kits. B, The expression of VLDLR in adipose tissues was observed by IHC. C, VLDLR protein levels were determined by western blot analysis using specific antibodies. β ‐actin was used as an internal control. The relative ratio level was determined by densitometric analysis. Values are represented as mean ± SD (n = 8); ^###^ *P* < .001 vs Con group; ***P < .001 vs HFD group; significances between treated groups were determined using ANOVA and Dunnett's post hoc test. D, The colocalization of VLDLR and iNOS in ATM from mice with HFD‐induced obesity was observed. Whole‐mount IF analysis demonstrated nucleus (blue), VLDLR (green) and iNOS (red) in adipose tissues from each experimental group. The right panels show merged images from the individual left and middle panels. Note that the yellow‐coloured regions indicate the co‐localization of the target molecules in the right panels. Scale bar = 25 μm

Figure 2A demonstrates that NK treatment suppressed the VLDLR mRNA expression in the 3T3‐L1 adipocytes. In addition, as shown in Figure 4D, NK significantly reduced the levels of serum triglyceride, causing us to question whether NK inhibits adipogenesis via the VLDL‐VLDLR signalling pathway in the HFD‐induced obesity mouse model. We measured the amount of intracellular VLDL and confirmed that the administration of NK attenuated the amount of VLDL in the adipose tissue of HFD‐induced obese mice (Figure 5A). As seen from Figure 5B, VLDLR protein levels in adipose tissues were higher in the HFD group than in the control group, whereas the administration of Orli and NK inhibited the overexpression of VLDLR in adipose tissues. A similar trend was observed in the immunoblotting experiments on VLDLR (Figure 5C). iNOS, a enzyme catalysing the production of nitric oxide, plays an important role in the development of adipose tissue inflammation.^31^ To investigate the inhibitory effect of NK on VLDLR, the extent of colocalization of VLDLR and iNOS was examined in adipose tissues obtained from mice with HFD‐induced obesity. Compared to the control group, the levels of fluorescence‐conjugated iNOS were elevated in the crown‐like structure in adipose tissues from the HFD group, whereas the administration of NK notably suppressed the overexpression of iNOS in ATMs. Moreover, elevated VLDLR fluorescence was detected in iNOS^+^ ATMs in the adipose tissues obtained from obese mice. However, as illustrated in the merged image, the overexpressed VLDLR in iNOS^+^ ATMs was suppressed by the administration of NK. These results imply that the administration of NK could suppress inflammatory responses in obese adipose tissues via the inhibition of VLDLR (Figure 5D).

### 3.8 NK inhibits obesity‐induced inflammation via the MAPK/ERK signalling pathway in the HFD‐induced obesity mouse model

**FIGURE 6:**
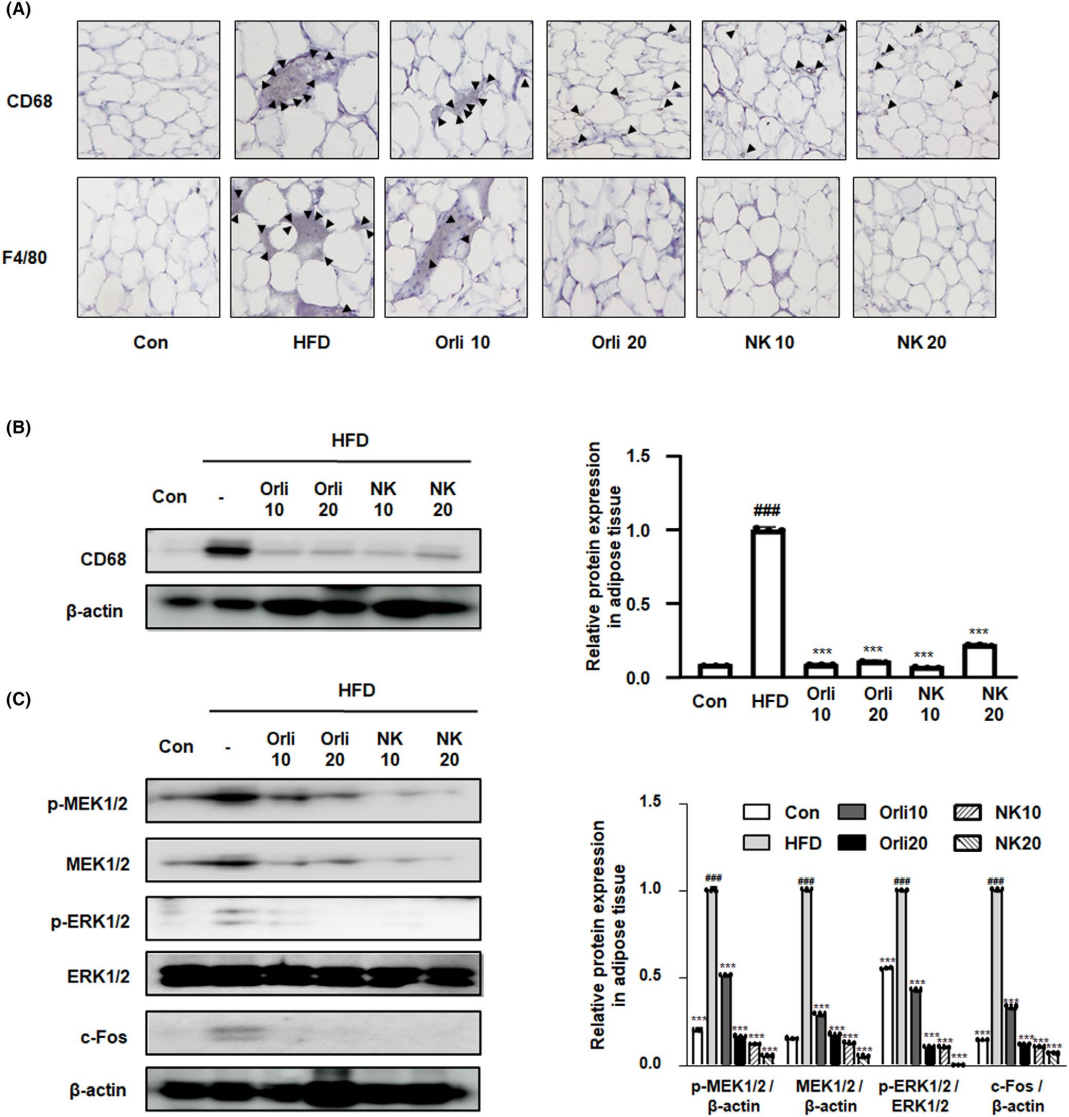
Effect of NK on obesity‐induced inflammation in mice with HFD‐induced obesity. A, The expression of CD68 and F4/80 in the adipose tissues was observed by IHC. Immunoblot results demonstrated the levels of (B) CD68 and (C) MEK/ERK/c‐Fos pathway in the adipose tissues. Densitometric protein levels of (B) CD68, (C) p‐MEK1/2, MEK1/2, p‐ERK and c‐Fos are represented as mean ± SD and plots of each protein are shown. β ‐actin and ERK were used as internal controls. ^###^ *P* < .001 vs Con group; ***P < .001 vs HFD group; significant differences between the treatment groups were determined using ANOVA and Dunnett's post hoc test

As shown in Figure 4B, the crown‐like structure formed by infiltrating macrophages was well‐developed in the HFD group compared to the phenotype in the control group, whereas the administration of NK alleviated its appearance, suggesting the inhibitory effects of NK on activated macrophages in adipose tissues. When immunostaining was conducted using anti‐CD68 and anti‐F4/80 antibodies, which may be oxidative stress attributable to macrophage markers; the accumulation of macrophage markers around the adipocytes was notably increased in the HFD group, whereas the administration of NK inhibited CD68 and F4/80 (Figure 6A). Western blot data also demonstrated that the protein expression of CD68 significantly decreased in the NK10 and NK20 groups compared to that in the HFD group (Figure 6B). Next, we examined whether MAPK/ERK‐dependent adipose tissue inflammation is regulated by the administration of NK. As shown in Figure 6C, NK administration significantly suppressed the obesity‐induced increase in MEK/ERK phosphorylation, as well as an increase in c‐Fos protein expression. These results corroborate the hypothesis stating that NK inhibits adipogenesis and obesity‐induced macrophage infiltration, simultaneously.

## 4 DISCUSSION

In this study, we utilized 3T3‐L1 adipocytes and HFD‐induced obese mice to evaluate the therapeutic effects of NK and the underlying mechanism of the anti‐adipogenic and anti‐inflammatory effects of NK. Following the differentiation of 3T3‐L1 cells that harbour most of the structural characteristics of adipocytes from animal tissue, the accumulation of lipid droplets is observed in the cells. In the results obtained from our in vitro experiments, the inhibitory effects of NK on the lipid droplet formation were elucidated by the significant reversal of expression of several lipid metabolism and adipocyte differentiation‐specific genes, including SREBP‐1, PPARγ, C/EBPα, C/EBPβ, FAS, HMG‐CoA, aP2, LXR, LDLR, VLDLR (Figure 1). The major adipogenic transcription factors, PPARγ and C/EBPα, enhance the expression of triglyceride synthesis genes, such as aP2 and FAS. Several studies have explored the role of SREBP and LXR in obesity and adipogenesis. LXRs regulate lipid metabolism by modulating the expression of lipogenesis genes (SREBP1 and FAS) and adipocyte‐specific genes (PPARγ and aP2).^32^, ^33^ SREBPs are highly expressed in adipose tissues and play a vital role in adipocyte development, including roles in the induction of PPARγ and the expression of several genes critical for lipid biosynthesis, such as HMG‐CoA.^34^ Meanwhile, the phosphorylated form of AMPK inhibits the expression of genes associated with lipid synthesis, including FAS and SREBP. AMPK activation imparts protection against HFD‐induced obesity via regulation of energy metabolism in adipose tissues. AMPK, in addition to the metabolic described above, also function in responding to oxidative stress and inflammation. Our experiments confirmed altered AMPK phosphorylation under differentiation‐inducing conditions compared to the control group. However, NK treatment restored the levels of AMPK phosphorylation, indicating that NK exerts anti‐adipogenic and antioxidant effects (Figure 1E).

In line with the results obtained from the in vitro study, the NK‐administered mice demonstrated significantly reduced weight gain and suppressed overproduction of fat pads compared to those in the mice belonging to the HFD group (Figures 3 and 4A). In addition, NK treatment notably suppressed HFD‐induced dyslipidaemia in the mouse model, where the inhibitory effects of NK on the high levels of triglyceride were more potent than those in the Orli‐administered group (Table 3). Consistent with these results, NK notably suppressed the hypertrophy and hyperplasia of adipocytes (Figure 4B,C). Th anti‐adipogenic effects of NK were also confirmed by the significant suppression of protein expression of the adipogenic transcription factors, including SREBP‐1, PPARγ and C/EBPα (Figure 4D). By interacting with the adipose tissue, liver tissue plays a vital role in lipid metabolism. The HFD‐driven aberrant metabolic process would damage normal hepatic function and lead to the upregulation of oxidative stress and inflammation in systemic circulation and lipid disorders.^35^ As shown in Table 3, we observed altered serum levels of AST and ALT, which are well‐known enzymatic parameters of liver damage, in the HFD group compared to those in the control group. However, the NK20 administration significantly reduced the levels of these enzymes. In addition, the liver tissue of mice in the HFD group turned pale. NK administration alleviated this symptom of hepatic fat accumulation. It has been observed that the activation of hepatic SREBP‐1, PPARγ and C/EBPα factors is associated with the upregulation of several lipid uptake‐associated proteins and the formation of lipid droplets under obese state.^36^, ^37^, ^38^ Our results from the gene expression studies indicated that the administration of NK significantly reduced the overexpression of SREBP‐1, PPARγ and C/EBPα in the liver compared to their levels in the HFD group (Figure 3H). Hence, we predict that NK may exert inhibitory effects on obesity‐induced metabolic complications, such as hepatic steatosis.

In our study, the anti‐adipogenic effects of NK were assumed to be involved in the inhibition of the VLDLR signalling pathway. In addition, our results demonstrate that the administration of NK remarkably suppressed the overexpression of VLDLR protein and reduced VLDL levels in adipose tissues (Figures 2 and 5). This finding was unexpected and suggests that VLDLR impacts the uptake of VLDL at different levels. Consistent with our results, previous studies have demonstrated that the uptake of fatty acid is not compromised by the expression of VLDLR. The suppression of apoE, which is the natural ligand of VLDLR, reduces adipocyte hypertrophy and VLDLR expression.^39^, ^40^ Meanwhile, our results from VLDLR siRNA transfection indicated that the knockdown of VLDLR partially shrank the effects of NK on adipocytes, suggesting that NK directly inhibits VLDLR signalling. Structure‐based molecular docking analysis demonstrated that NK is predicted to interact with VLDLR, where NK forms hydrogen bonds with VLDLR at residues Glu89, Thr91 and Arg100, simultaneously.

As explained in the introduction, it is clear that VLDLR actively participates in adipocyte hypertrophy, excess lipid accumulation and overproduction of oxidative stress.^13^ In contrast, previous studies have shown that VLDLR deficiency not only reduced the size of adipocytes and but also concurrently reduced the ATM content, which is indicative of the role of VLDLR in the association between adiposity and inflammatory action.^16^ It has been also noted that macrophage VLDLR‐mediated VLDL uptake might influence inflammatory responses, thereby potentiating adipose tissue inflammation and insulin resistance in obesity.^41^ In accordance with the aforementioned studies, results from our study demonstrated a strong and consistent association between VLDLR and macrophage‐mediated adipose tissue inflammation. Meanwhile, NK administration notably reduced the overexpression of VLDLR in iNOS^+^ ATMs compared to that in the HFD group, supporting the hypothesis indicating the inhibitory role of NK in obesity and obesity‐induced inflammation (Figure 5).

CD68 was utilized to identify total macrophage infiltration in human adipose tissues where total adipose tissue areas and degree of adipocyte hypertrophy were positively associated with gene expression of CD68.^42^ Our data demonstrated that macrophage factors CD68 and F4/80 were overexpressed in the HFD group, whereas these effects were abolished by administration with NK (Figure 6A,B). Subsequently, we also demonstrated the inhibitory effects of NK on the phosphorylation of MEK1/2 and ERK1/2 as well as on the overexpression of MEK1/2 and c‐Fos in obesity‐induced mice (Figure 6C). These observations are supported by previous studies that demonstrate targeting the ERK1 isoform in the regulation of adipocyte differentiation and HFD–induced obesity.^43^ Other studies have observed that regulation of phosphorylation of ERK1/2 in adipose tissue is associated with macrophage infiltration of adipose tissue. This action mediates pro‐inflammatory activation of adipocytes within the adipose tissue.^44^ In addition, it has conclusively been demonstrated that MAPK signalling cascades influence the regulation of pro‐inflammatory and oxidative stress pathways that are intimately connected with adipose tissue inflammation.^45^

The purpose of this study was to determine the pharmacological effects of NK on obesity and its complications, including hepatic steatosis and obesity‐induced inflammation. The evidence from this study suggests that NK is a more potent anti‐adipogenic candidate that modulates the expression of several genes associated with lipid metabolism and adipogenesis in 3T3‐L1 adipocytes. To the best of our knowledge, this is the first study that has documented the interaction between NK and VLDLR structure. NK administration also alleviated HFD‐induced obesity in the animal model via the inhibition of oxidative stress‐mediated adipogenesis and the VLDLR signalling pathway.

This study investigated that NK suppresses obesity via the inhibition of oxidative stress‐mediated adipogenesis and the VLDLR signalling pathway. Inhibitory effect of NK is superior to that of Orlistat, highlighting the potential of NK as a therapeutic agent for treatment of obesity and its complications. In addition, to the best of our knowledge, this is the first study that has documented the interaction between NK and VLDLR structure.

## CONFLICTS OF INTEREST

The authors declare no conflict of interest.

## AUTHOR CONTRIBUTIONS

B.‐RJ and H.‐JA conceived and designed the experiments. B.‐RJ performed the experiments and B.‐RJ analysed the data with H.‐JA, M.‐HL and H.‐JA supplied the reagents, materials and analysis tools. B.‐RJ, M.‐HL and H.‐JA wrote the paper. All authors read and approved the final manuscript.

## INFORMED CONSENT STATEMENT

Not applicable.

## Data Availability

The data that support the findings of this study are available from the corresponding author upon reasonable request.
